# Non-malaria fevers in a high malaria endemic area of Ghana

**DOI:** 10.1186/s12879-016-1654-4

**Published:** 2016-07-11

**Authors:** Kwaku Poku Asante, Seth Owusu-Agyei, Matthew Cairns, Ellen Boamah, Grace Manu, Mieks Twumasi, Richard Gyasi, George Adjei, Kingsley Kayan, Emmanuel Mahama, David Kwame Dosoo, Kwadwo Koram, Brian Greenwood, Daniel Chandramohan

**Affiliations:** Kintampo Health Research Centre, Ghana Health Service, P. O. Box, 200, Kintampo, Brong Ahafo Region Ghana; London School of Hygiene & Tropical Medicine, Keppel Street, London, WC1E 7HT UK; Department of Pathology, University of Ghana Medical School, College of Health, Accra, Ghana; Noguchi Memorial Institute for Medical Research, University of Ghana, Accra, P. O. Box LG 581, Ghana

**Keywords:** Malaria, Non malaria fevers, Fever epidemiology, Cohort study, Ghana

## Abstract

**Background:**

The importance of fevers not due to malaria [non–malaria fevers, NMFs] in children in sub-Saharan Africa is increasingly being recognised. We have investigated the influence of exposure-related factors and placental malaria on the risk of non-malaria fevers among children in Kintampo, an area of Ghana with high malaria transmission.

**Methods:**

Between 2008 and 2011, a cohort of 1855 newborns was enrolled and followed for at least 12 months. Episodes of illness were detected by passive case detection. The primary analysis covered the period from birth up to 12 months of age, with an exploratory analysis of a sub-group of children followed for up to 24 months.

**Results:**

The incidence of all episodes of NMF in the first year of life (first and subsequent) was 1.60 per child-year (95 % CI 1.54, 1.66). The incidence of NMF was higher among infants with low birth weight [adjusted hazard ratio (aHR) 1.22 (95 % CI 1.04–1.42) *p* = 0.012], infants from households of poor socio-economic status [aHR 1.22 (95 % CI 1.02–1.46) *p* = 0.027] and infants living furthest from a health facility [aHR 1.20 (95 % CI 1.01–1.43) *p* = 0.037]. The incidence of all episodes of NMF was similar among infants born to mothers with or without placental malaria [aHR 0.97 (0.87, 1.08; *p* = 0.584)].

**Conclusion:**

The incidence of NMF in infancy is high in the study area. The incidence of NMF is associated with low birth weight and poor socioeconomic status but not with placental malaria.

**Electronic supplementary material:**

The online version of this article (doi:10.1186/s12879-016-1654-4) contains supplementary material, which is available to authorized users.

## Background

The burden of malaria in sub-Saharan Africa is still high [1] although there are areas where it has fallen substantially in the past few years as a result of scaling up of coverage with insecticide treated nets (ITN), selective use of indoor residual spraying (IRS) and introduction of efficacious artemisinin-based combination therapy (ACT) [2–6]. The relatively recent introduction of rapid diagnostic tests for malaria has shown that a high proportion of fevers in children are due to other diseases, even in settings with high malaria transmission [7]. As a consequence of the reduction in malaria burden, and the wider use of diagnostics, the importance of fevers not due to malaria (non–malaria fevers, (NMFs)) in malaria endemic areas is gaining increasing recognition [8]. The causal agents of non-malaria fevers are diverse, vary from area to area and include protozoa, bacteria and viruses [9–11].

Some environmental and social factors are known to be associated with NMF. In a study undertaken in Nigeria, the risk of NMF was increased among the poor and among rural populations [12]. Poverty, high population density, poor water supply and climatic changes are associated with specific infections [13–16]. Another factor identified as a risk factor for NMFs in Benin, West Africa was exposure to malaria antigens in utero, although the mechanism through which this could have been brought about is currently unclear [17, 18].

To investigate the influence of social and environmental factors, placental malaria and gravidity on the risk of NMFs among young children, we have analysed data from the Kintampo Birth Cohort Study which was conducted between 2008 and 2011 in an area of Ghana with high malaria transmission [19]. This study provides further understanding of the epidemiology of NMF in a high malaria transmission area and provides basis of planning health interventions that target NMF.

## Methods

### Study area

A prospective birth cohort study to explore the relationship between placental malaria and malaria in infancy was conducted between 2008 and 2011 in the Brong-Ahafo region of Ghana; this study is described in detail elsewhere [19]. Malaria transmission in the study area is high (entomological inoculation rate - 269 infective bites/person/year) and perennial, but transmission peaks between April and October [20]. The health system in the study area is basic and includes public and private health facilities. Infant mortality rate is relatively high, estimated at 52 deaths per 1,000 live births in 2010 [21] and about 40 per 1000 live births in 2013 (Kintampo Health Research Centre, 2015 Report). Laboratory investigations for non-malarial infections are limited to bacterial cultures, which are available in only one health facility.

### Study procedures

The study procedures have been reported in detail elsewhere [19]. In summary, forty-two communities where good follow-up could be obtained were selected from within the Kintampo Health and Demographic Surveillance System (KHDSS). All pregnant women resident in the selected communities were identified using vital registers collated by community key informants or by staff of the KHDSS [21] who made home visits. At enrolment, demographic, socio-economic and obstetric characteristics of study women were recorded by trained fieldworkers using a standard questionnaire. Study women were followed throughout pregnancy until delivery and, whenever possible, a placental sample was obtained. The malaria status of the placenta was defined as showing either 1) an acute infection (parasites present with minimal pigment), 2) a chronic infection (parasites and substantial pigment present) 3) a past malaria infection (substantial pigment only) or 4) no evidence of malaria infection [22, 23].

### Enrolment and follow-up of study infants

Newborns of mothers who had been enrolled in the study prior to delivery were included in the infant cohort study. All infants recruited to the study (with the exception of those who died, migrated or were lost to follow-up) were followed for a minimum of 12 months. However, because recruitment to the study was gradual and children remained in follow-up until the end of the study in May 2011, some children were followed up to 24 months of age.

Episodes of illness were detected passively at study clinics. To maximise capture of fevers, families were provided with health insurance for the duration of the study and encouraged to attend clinics whenever an infant was unwell. Furthermore, community-based fieldworkers facilitated transportation of sick infants to see a study clinician for clinical evaluation. On evaluation by a study clinician, a history of fever within the 48 h prior to the clinic visit was recorded and an axillary temperature was measured with a digital thermometer.

Infants’ illnesses were investigated and managed according to the Ghana National Treatment Guidelines. Rapid diagnostic tests were used to diagnose malaria prior to treatment at the clinic. Thin and thick peripheral blood smears were also made and read subsequently, following the methods described by Swysen et al. [24]. Blood culture, serological or molecular assays for other infectious agents were not done routinely. Non-malaria fevers, the focus of this study, were defined as 1) the presence of fever (a history of fever in the last 48 h prior the clinic visit OR a measured axillary temperature ≥ 37.5 °C) and 2) no malaria parasitaemia detected by microscopy. The cause of death among the study cohort was assessed using verbal post mortem.

### Statistical analysis

Cleaned data were analyzed using STATA 13 (StataCorp, College Station, TX.). Principal component analysis of women’s durable assets was used to derive quintiles of socio-economic status (SES), as described previously [19, 25–27]. Cox regression models were used to determine hazard ratios for multiple episodes of NMF, using a robust standard error to account for within-child correlation. The Efron method was used for tied failure times. Potential risk factors including household characteristics: place of residence (urban, rural), household size (<5, 5–9, ≥10 residents), socio-economic status, roof construction (thatched or other); maternal characteristics: number of courses of intermittent preventive therapy in pregnancy (IPTp) received and infant characteristics: sex, birth weight (low, < 2.5 kg; normal), the season of birth (wet, April-November; dry, December-March), and ITN use. ITN use was assessed as tertiles (high, medium or low) based on scores of ITN use in the previous night of scheduled home visits made to access the presence of participants in the study area during follow up.

The primary analysis covered the period from birth up to the age of 12 months. A previous study of non-malaria fevers in Benin [18] investigated non-malaria fevers by subtype in children less than 18 months of age. Since respiratory and gastrointestinal diseases are common among young children, the analysis was repeated to determine the incidence of NMFs accompanied by gastrointestinal and respiratory symptoms., Since, depending on the date of enrolment, some children were followed for longer than 12 months, we also conducted exploratory analyses of incidence patterns in the period 0–18 months and 6–18 months of age as in the Benin study.

### Ethical approvals

The study was approved by the ethics committees of the Kintampo Health Research Centre (KHRC), Ghana Health Service, London School of Hygiene & Tropical Medicine and Noguchi Memorial Institute for Medical Research. Written informed consent was sought from all study women.

## Results

### Characteristics of study children

**Fig. 1:**
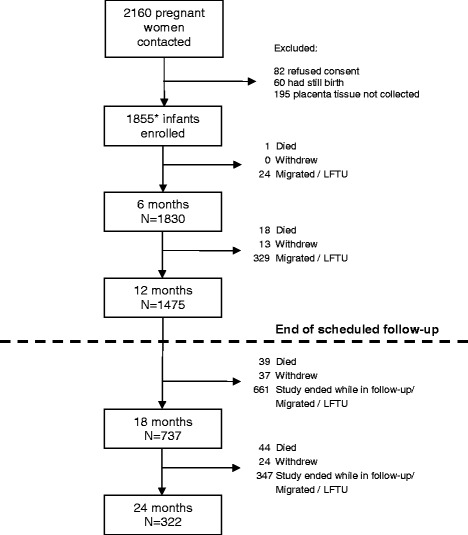
Study flow diagram of infants born into the Kintampo Birth Cohort Study. *The cohort included 72 children from twin pregnancies

A cohort of 1855 newborns was recruited and followed; 79.5 % (1475) completed the scheduled 1 year of follow up. Additionally, 39.5 % (737) and 17.4 % (322) were followed for 18 and 24 months respectively (Fig. 1). Six hundred and ninety-five infants (37.5 %) were born to mothers with placental malaria (PM) (PM + ve children), 355 (19 %) to primigravidae (PG) and 1050 (57 %) to mothers who had taken all three scheduled doses of IPTp (Table 1). The majority of infants (1465, 78.9 %) lived in rural areas. The prevalence of LBW was 40/508 (7.87 %) in the dry season and 130/1308 (9.9 %) in the wet season (*p* = 0.175). Socio-demographic characteristics such are place of residence, household size, or socioeconomic status were not statistically different among children who died and children who survived (Table 2). Perinatal deaths were the main cause of death among children with a known cause of deaths (Table 3).

**Table 1:** Characteristics of study cohort

	Attribute	*n* (%)
Household characteristics		
Place of residence	Urban	390 (21.0)
	Rural	1465 (78.9)
Household size	<5	937 (50.7)
	5–9	740 (40.1)
	10+	170 (9.2)
Socio-economic status	Least poor	408 (22.0)
	Less poor	380 (20.5)
	Poor	322 (17.4)
	More poor	387 (20.9)
	Very poor	358 (19.3)
Thatched roof	No	1312 (70.7)
	Yes	543 (29.3)
Animals in household	No	532 (28.7)
	Yes	1323 (71.3)
Distance from health centre	<1 km	776 (41.8)
	1–4.9 km	620 (33.4)
	5–7.9 km	288 (15.5)
	≥8 km	171 (9.2)
Maternal characteristics		
Gravidity	Primigravid	355 (19.1)
	Multigravid	1500 (80.9)
Number of IPTp	0	97 (5.2)
Courses	1	240 (12.9)
	2	468 (25.2)
	3	1050 (56.6)
Placental malaria	Infected	695 (37.5)
	Non-infected	1160 (62.5)
Infant characteristics		
Birth weight	Normal	1646 (90.6)
	Low birth Weight	170 (9.4)
Sex	Male	945 (50.9)
	Female	910 (49.1)
Season of birth	Dec–Mar	521 (28.1)
	Apr–Nov	1334 (71.9)
Bednet use	High	587 (33.3)
	Medium	589 (33.4)
	Low	588 (33.3)

**Table 2:** Characteristics of children who died in the study cohort

	Attribute	Died		*P*-value
Household characteristics		Yes	No	
Place of residence	Urban	23 (22.5)	367 (20.9)	0.70
	Rural	79 (77.5)	1386 (79.1)	
Household size	<5	54 (52.9)	883 (50.6)	0.29
	5–9	35 (34.3)	705 (40.4)	
	10+	13 (12.8)	157 (9.0)	
Socio-economic status	Least poor	20 (19.6)	388 (22.1)	0.82
	Less poor	23 (22.5)	357 (20.4)	
	Poor	15 (14.7)	307 (17.5)	
	More poor	21 (20.6)	366 (20.9)	
	Very poor	23 (22.6)	335 (19.1)	
Thatched roof	No	60 (58.8)	1252 (71.4)	0.01
	Yes	42 (41.2)	501 (28.6)	
Animals in household	No	32 (31.4)	500 (28.5)	0.54
	Yes	70 (68.6)	1253 (71.5)	
Distance from health centre	<1 km	35 (34.3)	741 (42.3)	0.26
	1–4.9 km	35 (34.3)	585 (33.4)	
	5–7.9 km	22 (21.6)	266 (15.2)	
	≥8 km	10 (9.8)	161 (9.2)

**Table 3:** Cause of death among the study cohort (*N* = 102)

Cause of Death	Number	Percent
Unknown	53	52.0
Perinatal death	24	23.5
Septicaemia	10	9.8
Respiratory infection	4	3.9
Diarrhoea	3	2.9
Meningitis	2	2.0
Anaemia	1	1.0
Congenital Malformation	1	1.0
Malaria	1	1.0
Malnutrition	1	1.0
Parasitic disease	1	1.0
Unknown infection	1	1.0

### Incidence of NMFs

**Fig. 2:**
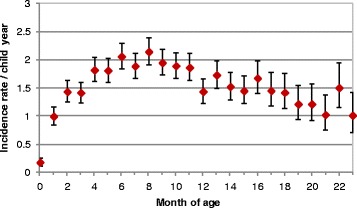
Incidence of non-malaria fevers during infancy; 95 % confidence interval for the rates are shown

A total of 2838 episodes of NMF were recorded during the first 12 months of life, 1302 (45.9 %) of which were associated with respiratory symptoms, 911 (32.1 %) with gastro-intestinal symptoms, and 625 (22.0 %) with other symptoms. During the same period of follow-up, 1242 febrile malaria episodes were recorded. Sixty-five percent (1205/1855) of the study children experienced at least one episode of NMF. The incidence of all (first and subsequent) episodes of NMF in infancy was 1.60 per child-year (95 % CI 1.54, 1.66). The incidence of NMF was low in early infancy, and rose quickly to around two episodes per child-year between 6 and 8 months of age, after which the incidence reached a plateau (Fig. 2).

### Risk factors for NMFs

The incidence of NMF between 0 and 12 months of age was higher in poor children, with incidence elevated in all SES categories relative to the most wealthy group (Table 4). The incidence of NMF was higher among children born during the rainy season (between April and November) than in those born during the dry season [unadjusted hazard ratio (uHR) 1.42 (95 % CI 1.26–1.60, *p* < 0.001); adjusted hazard ratio (aHR) 1.39 (95 % CI 1.23–1.56, *p* < 0.001)], higher among children who had a low birth weight compared with children who had normal birth weight [uHR 1.15 (95 % CI 0.97–1.35, *p* = 0.100); aHR 1.22 (95 % CI 1.04–1.42), *p* = 0.012], higher among those living furthest (>8Km) from the nearest health facility [uHR 1.30 (95 % CI 1.09–1.54, *p* = 0.003); aHR 1.20 (95 % CI 1.01–1.43, *p* = 0.037)] and, unexpectedly, higher among those whose mothers had used three courses of IPTp [uHR 1.43 (95 % CI 1.12–1.81, *p* = 0.004); aHR 1.31 (1.03, 1.68, *p* = 0.030)] (Table 4). There was a trend towards an increased risk for each additional course of IPTp taken: aHR 1.12, (1.06, 1.19; *p* < 0.001). The incidence of NMF was lower among girls than boys [uHR 0.90 (95 % CI 0.81–0.99, *p* = 0.033); aHR 0.87 (95 % CI 0.79–0.96) *p* = 0.006] and among children who used their bednet less regularly [uHR 1.13 (95 % CI 1.00–1.28, *p* = 0.075); aHR 1.15 (95 % CI 1.02–1.31) *p* = 0.024].

**Table 4:** Hazard ratio for all non malaria fevers with 0–12 months of follow up

Risk factor		Unadjusted HR (95 % CI)	*p*-value	Adjusted HR (95 % CI)	*p*-value
Household factors					
Place of residence	Urban	-	-	-	-
	Rural	1.26 (1.11, 1.42)	<0.001	0.94 (0.78, 1.14)	0.519
Household size	<5	-	-	-	-
	5–9	1.06 (0.95, 1.18)	0.299	1.03 (0.92, 1.14)	0.632
	10+	1.17 (0.97, 1.42)	0.098	1.10 (0.91, 1.32)	0.320
Socio-economic	Least poor	-	-	-	-
status	Less poor	1.31 (1.13, 1.54)	0.001	1.25 (1.07, 1.46)	0.005
	Poor	1.40 (1.18, 1.65)	<0.001	1.22 (1.02, 1.46)	0.027
	More poor	1.59 (1.36, 1.86)	<0.001	1.46 (1.23, 1.72)	<0.001
	Very poor	1.36 (1.15, 1.60)	<0.001	1.26 (1.05, 1.52)	0.012
Thatched roof	No	-	-	-	-
	Yes	1.21 (1.08, 1.35)	0.001	1.10 (0.97, 1.24)	0.128
Animals in household	No	-	-	-	-
	Yes	1.14 (1.02, 1.29)	0.023	1.06 (0.95, 1.19)	0.307
Distance from health	<1 km	-	-	-	-
centre	1–4.9 km	0.83 (0.74, 0.94)	0.002	0.90 (0.76, 1.06)	0.204
	5–7.9 km	1.19 (1.02, 1.38)	0.025	1.19 (1.03, 1.37)	0.019
	≥8 km	1.30 (1.09, 1.54)	0.003	1.20 (1.01, 1.43)	0.037
Maternal factors					
Gravidity	Primigravid	-	-	-	-
	Multigravid	0.94 (0.83, 1.07)	0.377	0.94 (0.82, 1.07)	0.367
Number of IPTp	0	-	-	-	-
courses	1	1.16 (0.88, 1.53)	0.306	1.03 (0.77, 1.36)	0.862
	2	1.28 (1.00, 1.65)	0.055	1.11 (0.86, 1.44)	0.432
	3	1.43 (1.12, 1.81)	0.004	1.31 (1.03, 1.68)	0.030
Placental infection	Uninfected	-	-	-	-
	Infected	1.04 (0.93, 1.15)	0.516	0.97 (0.87, 1.08)	0.584
Infant factors					
Birth weight	Normal	-	-	-	-
	Low birth weight	1.15 (0.97, 1.35)	0.100	1.22 (1.04, 1.42)	0.012
Sex	Male	-	-	-	-
	Female	0.90 (0.81, 0.99)	0.033	0.87 (0.79, 0.96)	0.006
Season of birth	Dec–Mar	-	-	-	-
	Apr–Nov	1.42 (1.26, 1.60)	<0.001	1.39 (1.23, 1.56)	<0.001
Bednet use	High	-	-	-	-
	Medium	1.14 (1.01, 1.28)	0.032	1.16 (1.03, 1.30)	0.015
	Low	1.13 (1.00, 1.28)	0.053	1.15 (1.02, 1.31)	0.024

The incidence of NMF between 0 and 12 months of age among children born to mothers without placental malaria was very similar to that observed among children born to mothers who had placental malaria [uHR 1.04 (95 % CI 0.93–1.15, *p* = 0.516), aHR 0.97 (95 % CI 0.87–1.08, *p* = 0.584)] (Table 4). The incidence of NMF among children born to primigravidae was very similar to that among children born to multigravidae [uHR 0.94 (95 % CI 0.83–1.07), *p* = 0.377, aHR 0.94 (95 % CI 0.82–1.07) *p* = 0.367] (Table 4). There was no evidence for an interaction between placental malaria and gravidity on the risk of non malaria fevers (Wald test, *p* = 0.79).

In exploratory analyses making use of the children who were followed for a longer period, there was no difference in the incidence of NMF in children born to mothers with or without PM between 0 and 18 months of age [uHR 1.01 (95 % CI 0.92–1.12), *p* = 0.816), aHR 0.95 (95 % CI 0.87–1.05, *p* = 0.349)] (Additional file 1: Table S1) or between 6 and 18 months of age [uHR 1.01 (95 % CI 0.91–1.13), *p* = 0.808, aHR 0.96 (95 % CI 0.86–1.07, *p* = 0.418)] (Additional file 2: Table S2). There were also no differences by gravidity over the longer period of follow-up.

### Factors associated with NMF and respiratory symptoms

The factors associated with NMFs accompanied by respiratory symptoms were similar to those associated with all NMFs. Between 0 and 12 months of age, the incidence of respiratory NMFs was higher among those of low birth weight, those born during the rainy season and among those of more poor socioeconomic status (Table 5). There was also a suggestion that girls were at slightly lower risk of respiratory fevers during infancy than boys. There were no differences in the incidence of NMFs associated with respiratory symptoms between children born to mothers with or without placental malaria (Table 5).

**Table 5:** Adjusted hazard ratio (aHR) for all episodes of fever associated with respiratory or gastrointestinal (GI) symptoms between 0 and 12 months of age

Risk factor		Respiratory aHR (95 % CI)	*p*-value	GI aHR (95 % CI)	*p*-value
Household factors					
Place of residence	Urban	-	-	-	-
	Rural	0.91 (0.70, 1.17)	0.442	1.02 (0.74, 1.41)	0.884
Household size	<5	-	-	-	-
	5–9	1.02 (0.89, 1.17)	0.729	0.89 (0.76, 1.06)	0.186
	10+	1.08 (0.85, 1.37)	0.541	1.36 (1.05, 1.77)	0.021
Socio-economic	Least poor	-	-	-	-
status	Less poor	1.23 (1.00, 1.51)	0.050	1.38 (1.06, 1.80)	0.015
	Poor	1.30 (1.03, 1.65)	0.027	1.28 (0.97, 1.69)	0.083
	More poor	1.34 (1.08, 1.66)	0.008	1.47 (1.12, 1.94)	0.006
	Very poor	1.15 (0.91, 1.46)	0.248	1.46 (1.10, 1.95)	0.009
Thatched roof	No	-	-	-	-
	Yes	1.05 (0.89, 1.23)	0.575	1.15 (0.96, 1.38)	0.121
Animals in household	No	-	-	-	-
	Yes	0.99 (0.86, 1.14)	0.908	1.13 (0.94, 1.35)	0.191
Distance from health	<1 km	-	-	-	-
centre	1–4.9 km	0.92 (0.74, 1.15)	0.464	0.90 (0.68, 1.18)	0.430
	5–7.9 km	1.25 (1.04, 1.50)	0.017	1.30 (1.05, 1.61)	0.015
	≥8 km	1.06 (0.82, 1.36)	0.649	1.51 (1.17, 1.94)	0.001
Maternal factors					
Gravidity	Primigravid	-	-	-	-
	Multigravid	1.05 (0.88, 1.24)	0.592	0.83 (0.68, 1.00)	0.052
Number of IPTp	0	-	-	-	-
courses	1	1.14 (0.77, 1.70)	0.509	1.14 (0.70, 1.85)	0.596
	2	1.12 (0.78, 1.61)	0.539	1.28 (0.82, 2.01)	0.273
	3	1.31 (0.93, 1.86)	0.125	1.67 (1.08, 2.59)	0.022
Placental infection	Uninfected	-	-	-	-
	Infected	0.95 (0.82, 1.10)	0.529	0.97 (0.82, 1.13)	0.668
Infant factors					
Birth weight	Normal	-	-	-	-
	Low birth weight	1.27 (1.03, 1.57)	0.025	1.22 (0.96, 1.55)	0.104
Sex	Male	-	-	-	-
	Female	0.87 (0.76, 0.98)	0.027	0.98 (0.84, 1.15)	0.832
Season of birth	Dec–Mar	-	-	-	-
	Apr–Nov	1.51 (1.29, 1.76)	<0.001	1.54 (1.28, 1.85)	<0.001
Bednet use	High	-	-	-	-
	Medium	1.15 (0.98, 1.33)	0.081	0.97 (0.80, 1.17)	0.724
	Low	1.11 (0.95, 1.31)	0.195	1.04 (0.87, 1.26)	0.643

### Factors associated with NMFs and gastrointestinal symptoms

The incidence of NMFs associated with gastrointestinal symptoms between 0 and 12 months of age was higher among children from larger households, those from more poor or very poor families, those living far from a health centre and those born during the rainy season (Table 5). As in the main analysis, incidence was higher in children whose mothers had received three doses of IPTp, There was again no difference in the incidence of gastrointestinal NMFs according to placental malaria status (Table 5).

## Discussion

We followed a cohort of 1855 children during infancy, and investigated the relationship between social and environmental factors, placental malaria and gravidity on the incidence of non-malaria fevers (NMFs). Our findings indicate that despite the well-documented high burden of malaria in this setting, non-malaria fevers contribute a larger fraction of the febrile disease burden in the first year of life.

Since cases of NMF were identified passively and therefore dependent on the health seeking behaviour of mother or caregivers of the infants, it is likely that the true incidence of NMF is higher still. It is possible that children living in this high malaria transmission area are predisposed to other febrile illness due to the modulation of the immune system [28–30] that results from repeated malaria infections, as reported previously [31, 32].

The high burden of NMFs in young children is concentrated predominantly among poor families living far from health facilities. Children with low birth weight were found to be at an increased risk of NMFs over the course of the study. Low birth weight infants are known to be at a higher risk of bacterial and viral infections [33], possibly due to their immature immune systems as observed among preterm babies [34]. Male children were at a higher risk of having NMF compared to females. Gender differences in disease epidemiology have been well documented among adults and to a lesser extent among children [35]. The risk of bacterial infections and infant mortality are higher in boys than girls [36–38]. These gender differences may be due to genetic and hormonal differences between boys and girls that may be applicable to our study cohort [39]. However, there may also be differences in care seeking behaviour for illnesses in boys or girls. The incidence of NMF was higher among children born in the long wet season (April–November) compared to children born in the dry season. Birth in the wet season is associated with infections such as respiratory syncytial virus infections [40]. Although we attempted to control for this in a multivariable model, it is also possible that the higher incidence of NMF among infants born during the rainy season may be related to the higher risk of LBW among those born in the wet season.

The finding that NMFs were more frequent in children born to women who had received several courses of IPTp was unexpected. This result was seen in different sub-categories of NMFs, and remained after adjustment for a number of possible confounders including distance from a health centre, place of residence and SES. It also remained in exploratory analyses over a longer period of follow-up. One possibility is that mothers who received IPTp were especially conscientious about seeking care for their children when they were ill, resulting in higher apparent incidence (i.e. the apparent link between higher IPTp use and NMF incidence is a result of confounding by care-seeking/access to care). However, if this was the case, it might also be expected that the children of these mothers would have an increased incidence of malaria; this was not found in our previous analysis of children in this cohort [19]. It is difficult to suggest a mechanism by which IPTp could increase the risk of non-malaria fevers, without increasing risk of malaria and consequently this may be a chance finding, or attributable to residual confounding; this should be examined in future studies.

NMFs were no more frequent in children born to women with placental malaria at delivery than in children whose mothers did not have placental malaria either in crude or adjusted analyses. There was also no association between the incidence of NMFs and mothers’ gravidity. In a previous study carried out in Benin, placental malaria infection was associated with a higher risk of NMFs among young children [18] and the investigators suggested that exposure to malaria antigens in utero predisposes young infants to NMF [18], as has been suggested previously for malaria [17]. The increased incidence of malaria among infants born to mothers with placental malaria (PM) in relatively low malaria transmission areas [41–45] could be due to exposure of these infants to malaria antigens in utero leading to changes in the immune system which make them more susceptible to malaria [17]. Alternatively, mothers with placental malaria may be at increased risk of malaria due to a combination of social and environmental risks which may be shared with their infants [19]. These social and environment factors could also increase the risk of NMFs in infants born to women who had placental malaria.

The reason for differences between our results and those of the study in Benin is unknown, although we performed analyses to match those undertaken in the Benin study as closely as possible. There were however some methodological differences between the two studies. Firstly, the transmission of malaria in Kintampo was much higher than in Tori Bossito, Benin (entomological inoculation rate (EIR) 269 infectious bites per person per year [20] and 20.5 respectively) [42, 43]. Thus, levels of in utero exposure to malaria may be higher in Kintampo than in Tori Bossito. Secondly, we used placental histology to diagnose placental malaria, a more sensitive test for placental malaria than the use of placental blood smears [46–48], the technique employed in Benin. Therefore we may have detected placental infections in this study that would have been missed in the previous study [18]. This could have biased results in the earlier study if high density infections acquired more recently were more likely to be detected, since this would tend to identify women with higher exposure to malaria (who may therefore be poorer, live in rural areas, etc., and therefore be more susceptible to malaria and NMFs). Finally, more children were followed in the present study than in the study undertaken in Benin. Although it was not possible to observe all study children until 18 months of age, exploratory analyses restricted to those who were followed to 18 months of age and beyond indicated very similar results to our analyses focusing on infancy.

The following potential biases are acknowledged in this study. Firstly, NMF cases were passively detected, therefore the true incidence may have been under-estimated depending on the health seeking behaviour of caregivers. To address this, care-givers were provided with free transportation and health insurance, and encouraged to attend study clinics whenever their child was unwell. Secondly, since there is no diagnostic test routinely carried out for the non-malarial causes of fever, it is possible that co-infections of malaria and other causes of fever such as viral infections were misclassified as malaria-only infections. This may also have led to underestimation of the incidence of NMF. Similarly the incidence of NMF may be underestimated if mortality among the study participants were as a result of NMF that were not reported.

Despite these issues that may lead to an underestimate of malaria incidence, and despite the high level of malaria transmission in this area of Ghana, the incidence of NMF is high. This underscores the importance of other causes of fever in many settings where malaria has previously been the major focus. There is an urgent need to determine the most frequent causes of NMFs in different ecological situations to allow the development of therapeutic and preventative strategies based on sound scientific evidence.

## Conclusion

The incidence of NMF is high in the middle belt of Ghana. The incidence of NMF is associated with low birth weight and poor socioeconomic status but not with placental malaria.

## Abbreviations

ACT, artemisinin-based combination therapy; aHR, adjusted hazard ratio; CI, confidence interval; EIR, entomological inoculation rate; IPTp, intermittent preventive treatment in pregnancy; IRS, indoor residual spraying; ITN, Insecticide treated nets; KHDSS, Kintampo Health and Demographic Surveillance System; KHRC, Kintampo Health Research Centre; LBW, low birth weight; MG, multigravidae; NMF, non malaria fevers; PG, primigravidae; PM, placental malaria; SES, socio-economic status; uHR, unadjusted hazard ratio

## Additional files

Additional file 1: Table S1. Hazard ratio for all episodes of NMF between 0 and 18 months of age. (DOC 77 kb) Additional file 2: Table S2. Hazard ratio for all episodes of NMF between 6 and 18 months of age. (DOC 77 kb)
