# Supplementary material for: Non-malaria fevers in a high malaria endemic area of Ghana
Source: BMC Infect Dis. 2016 Jul 11;16:327. doi: 10.1186/s12879-016-1654-4 (PMC4940727; doi:10.1186/s12879-016-1654-4)
Supplement: Additional file 1: Table S1. — Hazard ratio for all episodes of NMF between 0 and 18 months of age. (DOC 77 kb) [file 12879_2016_1654_MOESM1_ESM.doc]

Additional file 1: Table S1: Hazard ratio for all episodes of NMF between 0 and 18 months of age.

| **Risk factor** |  | **Unadjusted HR (95% CI)** | **p-value** | **Adjusted HR (95% CI)** | **p-value** |
| --- | --- | --- | --- | --- | --- |
| **Household factors** |  |  |  |  |  |
| Place of residence | urban | - |  | - | - |
|  | rural | 1.30 (1.16, 1.47) | <0.001 | 0.95 (0.80, 1.14) | 0.612 |
| Household size | <5 | - |  | - | - |
|  | 5-9 | 1.06 (0.96, 1.17) | 0.237 | 1.02 (0.92, 1.12) | 0.738 |
|  | 10+ | 1.13 (0.95, 1.35) | 0.155 | 1.06 (0.89, 1.25) | 0.525 |
| Socio-economic | least poor | - |  | - | - |
| status | less poor | 1.29 (1.11, 1.50) | 0.001 | 1.22 (1.06, 1.42) | 0.007 |
|  | poor | 1.40 (1.20, 1.63) | <0.001 | 1.21 (1.03, 1.42) | 0.024 |
|  | more poor | 1.52 (1.31, 1.76) | <0.001 | 1.36 (1.16, 1.58) | <0.001 |
|  | very poor | 1.30 (1.11, 1.52) | 0.001 | 1.19 (1.01, 1.41) | 0.040 |
| Thatched roof | no | - |  | - | - |
|  | yes | 1.18 (1.07, 1.31) | 0.001 | 1.07 (0.96, 1.19) | 0.231 |
| Animals in household | no | - |  | - | - |
|  | yes | 1.19 (1.07, 1.33) | 0.002 | 1.10 (0.99, 1.23) | 0.079 |
| Distance from health | < 1 km | - |  | - | - |
| centre | 1-4.9 km | 0.78 (0.70, 0.88) | <0.001 | 0.83 (0.71, 0.97) | 0.021 |
|  | 5-7.9km | 1.12 (0.98, 1.29) | 0.100 | 1.12 (0.98, 1.28) | 0.088 |
|  | > 8 km | 1.25 (1.07, 1.45) | 0.004 | 1.18 (1.01, 1.37) | 0.033 |
| **Maternal factors** |  |  |  |  |  |
| Gravidity | primigravid | - |  | - | - |
|  | multigravid | 0.96 (0.85, 1.080 | 0.506 | 0.94 (0.83, 1.07) | 0.341 |
| Number of IPTp | 0 | - |  | - | - |
| courses | 1 | 1.15 (0.89, 1.48) | 0.278 | 1.03 (0.80, 1.32) | 0.820 |
|  | 2 | 1.35 (1.08, 1.70) | 0.009 | 1.20 (0.95, 1.50) | 0.124 |
|  | 3 | 1.48 (1.20, 1.84) | <0.001 | 1.38 (1.11, 1.72) | 0.004 |
| Placental infection | uninfected | - |  | - | - |
|  | infected | 1.01 (0.92, 1.12) | 0.816 | 0.95 (0.87, 1.05) | 0.349 |
| **Infant factors** |  |  |  |  |  |
| Birth weight | normal | - |  | - | - |
|  | low birth weight | 1.11 (0.95, 1.30) | 0.176 | 1.19 (1.03, 1.38) | 0.017 |
| Sex | male | - |  | - | - |
|  | female | 0.91 (0.83, 0.99) | 0.050 | 0.89 (0.81, 0.97) | 0.008 |
| Season of birth | Dec-Mar | - |  | - | - |
|  | Apr-Nov | 1.31 (1.17, 1.46) | <0.001 | 1.27 (1.15, 1.41) | <0.001 |
| Bednet use | high | - |  | - | - |
|  | medium | 1.14 (1.02, 1.27) | 0.019 | 1.17 (1.05, 1.30) | 0.005 |
|  | low | 1.12 (0.99, 1.25) | 0.057 | 1.16 (1.03, 1.30) | 0.013 |
