# Supplementary material for: Non-malaria fevers in a high malaria endemic area of Ghana
Source: BMC Infect Dis. 2016 Jul 11;16:327. doi: 10.1186/s12879-016-1654-4 (PMC4940727; doi:10.1186/s12879-016-1654-4)
Supplement: Additional file 2: Table S2. — Hazard ratio for all episodes of NMF between 6 and 18 months of age. (DOC 77 kb) [file 12879_2016_1654_MOESM2_ESM.doc]

Additional file 2: Table S2. Hazard ratio for all episodes of NMF between 6 and 18 months of age.

| **Risk factor** |  | **Unadjusted HR (95% CI)** | **p-value** | **Adjusted HR (95% CI)** | **p-value** |
| --- | --- | --- | --- | --- | --- |
| **Household factors** |  |  |  |  |  |
| Place of residence | urban | - |  | - | - |
|  | rural | 1.34 (1.18, 1.54) | <0.001 | 0.94 (0.76, 1.16) | 0.543 |
| Household size | <5 | - |  | - | - |
|  | 5-9 | 1.04 (0.93, 1.16) | 0.510 | 0.98 (0.88, 1.10) | 0.775 |
|  | 10+ | 1.08 (0.89, 1.31) | 0.413 | 1.00 (0.82, 1.20) | 0.960 |
| Socio-economic | least poor | - |  | - | - |
| status | less poor | 1.26 (1.06, 1.50) | 0.008 | 1.18 (0.99, 1.39) | 0.061 |
|  | poor | 1.36 (1.14, 1.62) | 0.001 | 1.14 (0.96, 1.36) | 0.142 |
|  | more poor | 1.43 (1.21, 1.69) | <0.001 | 1.24 (1.04, 1.48) | 0.014 |
|  | very poor | 1.20 (1.01, 1.43) | 0.041 | 1.09 (0.90, 1.31) | 0.382 |
| Thatched roof | no | - |  | - | - |
|  | yes | 1.16 (1.03, 1.30) | 0.014 | 1.06 (0.93, 1.20) | 0.380 |
| Animals in household | no | - |  | - | - |
|  | yes | 1.25 (1.10, 1.41) | <0.001 | 1.16 (1.02, 1.31) | 0.021 |
| Distance from health | < 1 km | - |  | - | - |
| centre | 1-4.9 km | 0.72 (0.64, 0.82) | <0.001 | 0.74 (0.61, 0.88) | 0.001 |
|  | 5-7.9km | 1.08 (0.93, 1.26) | 0.334 | 1.08 (0.93, 1.26) | 0.324 |
|  | > 8 km | 1.17 (0.99, 1.40) | 0.069 | 1.13 (0.94, 1.35) | 0.182 |
| **Maternal factors** |  |  |  |  |  |
| Gravidity | primigravid | - |  | - | - |
|  | multigravid | 0.96 (0.84, 1.10) | 0.589 | 0.94 (0.81, 1.08) | 0.349 |
| Number of IPTp | 0 | - |  | - | - |
| courses | 1 | 1.10 (0.83, 1.46) | 0.502 | 0.98 (0.74, 1.29) | 0.877 |
|  | 2 | 1.34 (1.04, 1.72) | 0.024 | 1.19 (0.92, 1.53) | 0.183 |
|  | 3 | 1.53 (1.21, 1.94) | <0.001 | 1.41 (1.11, 1.80) | 0.005 |
| Placental infection | uninfected | - |  | - | - |
|  | infected | 1.01 (0.91, 1.13) | 0.808 | 0.96 (0.86, 1.07) | 0.418 |
| **Infant factors** |  |  |  |  |  |
| Birth weight | normal | - |  | - | - |
|  | low birth weight | 1.04 (0.86, 1.26) | 0.670 | 1.13 (0.94, 1.35) | 0.192 |
| Sex | male | - |  | - | - |
|  | female | 0.91 (0.82, 1.01) | 0.073 | 0.89 (0.81, 0.99) | 0.029 |
| Season of birth | Dec-Mar | - |  | - | - |
|  | Apr-Nov | 1.24 (1.10, 1.40) | <0.001 | 1.21 (1.08, 1.36) | 0.001 |
| Bednet use | high | - |  | - | - |
|  | medium | 1.15 (1.01, 1.30) | 0.033 | 1.19 (1.05, 1.35) | 0.007 |
|  | low | 1.12 (0.98, 1.27) | 0.099 | 1.18 (1.04, 1.35) | 0.012 |
